# *Urtica dioica* Extract Abrogates Chlorpyrifos-Induced Toxicity in Zebrafish Larvae

**DOI:** 10.3390/ijms25126631

**Published:** 2024-06-16

**Authors:** Kamel Mhalhel, Yassine Kadmi, Ahlem Ben Chira, Maria Levanti, Lidia Pansera, Marzio Cometa, Mirea Sicari, Antonino Germanà, Marialuisa Aragona, Giuseppe Montalbano

**Affiliations:** 1 Zebrafish Neuromorphology Lab, Department of Veterinary Sciences, University of Messina, 98168 Messina, Italy; mblevanti@unime.it (M.L.); lidia.pansera@studenti.unime.it (L.P.); marzio.cometa@unime.it (M.C.); mirea.sicari@studenti.unime.it (M.S.); antonino.germana@unime.it (A.G.); marialuisa.aragona@unime.it (M.A.); 2 LASIRE, Equipe Physico-Chimie de l’Environnement, CNRS UMR 8516, Université Lille, Sciences et Technologies, CEDEX, 59655 Villeneuve d′Ascq, France; yassine.kadmi@univ-lille.fr; 3 Department of Chemistry, Université d’Artois, IUT de Béthune, 62400 Béthune, France; 4 LR22ES01 Laboratory of Biomathematics, Faculty of Sciences of Sfax, Department of Mathematics, P.O. Box 1171, Sfax 3000, Tunisia; chiraahlem@gmail.com

**Keywords:** *Urtica dioica*, nettle, chlorpyrifos, organophosphate pesticides, developmental toxicity, neuroprotection, natural compounds

## Abstract

Chlorpyrifos (CPF) is a widely used organophosphate insecticide, though its excessive use causes environmental contamination, raising concerns about its adverse effects on human health. In this regard, *Urtica dioica* stands out as a promising candidate for counteracting chemical ‘contaminant’ toxicity thanks to its therapeutic properties. Therefore, our study aimed to investigate the potential of an *Urtica dioica* ethanolic extract (UDE) to mitigate chlorpyrifos-induced toxicity. Eight compounds in the *Urtica dioica* ethanolic extract have been identified, most of which present significant potential as antioxidant, anti-inflammatory, and neuroprotective agents. Chlorpyrifos exposure altered hatching rates, increased the incidence of teratogenic effects, and upregulated the expression of brain-derived neurotrophic factor (Bdnf) in zebrafish larvae telencephalon. On the other hand, *UDE* demonstrated a preventive effect against CPF-induced teratogenicity, which is expressed by a lower morphological deformity rate. Moreover, the UDE showed a rather protective effect, maintaining the physiological condition of the telencephalon. Additionally, CPF altered the locomotor behavior of larvae, which was characterized by irregular swimming and increased activity. This defective behavioral pattern was slightly attenuated by the UDE. Our findings suggest that the UDE possesses significant protective properties against CPF-induced toxicity, probably conferred by its natural antioxidant and anti-inflammatory contents. Still, further research is needed to elucidate the recruited mechanisms and implicated pathways on UDE’s protective effects.

## 1. Introduction

CPF is a well-known organophosphate insecticide, acaricide, and miticide with the chemical name *O*,*O*-diethyl-*O*-(3,5,6-trichloro-2-pyridinyl) phosphorothionate (CAS No. 2921-89-2). It is used to prevent and control crop pests and diseases, though its excessive use causes environmental pollution.

The continuous and excessive use of CPF in recent decades has already led to widespread environmental contamination. Indeed, it is commonly monitored in soils, ground water, and surface water, as well as in solid and liquid dietary samples. Thus, there is no doubt that the misuses of CPF and other organophosphate pesticides can have adverse effects on non-target organisms, including humans [1].

In the European Union, chlorpyrifos has been prohibited from being marketed for use as an active substance in plant protection products since 2020 (Commission Implementing Regulation (EU) 2020/18 of 10 January 2020). In the USA, the US Environmental Protection Agency’s tolerance for chlorpyrifos for products with registered food uses was expired in 2022 [2].

Although CPF has been seriously limited in use in the United States and the EU, pesticide manufacturing and sales to countries that have not yet banned it continue.

Being from the same family of chemicals as the sarin nerve gas agent, CPF functions by attacking the nervous system [3]. Indeed, the central and peripheral nervous systems are the primary target organs for CPF toxicity due to the ability of the chlorpyrifos-oxon metabolite to inhibit the enzyme activities of acetylcholinesterase (AChE) and butyrylcholinesterase (BuChE), the neurotransmitter choline ester catalyzers, which terminate neurotransmission at cholinergic synapses [4].

From a detailed evaluation of the literature conducted by Eaton et al. [4], repeated exposure to chlorpyrifos at a daily dose of less than 14 μg/kg/day has little or no effect on either acetyl or butyryl cholinesterase activity in target tissue in adults. Thus, repeated exposure to chlorpyrifos at a daily dose of less than ~10 μg/kg/day would not be expected to have discernable effects on the enzyme activity of the target tissue AChE or BuChE [4]. At doses less than those causing frank neurological effects, chlorpyrifos do not show significant toxicity in organ systems other than the nervous system. Moreover, it is not considered to be teratogenic at doses that do not cause frank maternal toxicity [4].

Alternative mechanisms of action for CPF, other than the primary mechanism of inhibiting the enzyme activity of acetylcholinesterase (AChE) and butlycholinesterase (BuChE), could potentially contribute to toxic effects occurring in vivo at doses less than those that would induce the primary mechanism; thus, there is a necessity to further investigate the different mechanisms of action for CPF.

Moreover, it is well known that biologically active compounds found in medicinal plants can enhance neurological function through multiple pathways [5]. Among these plants, *Urtica dioica*, known as nettle, stands out for its remarkable medicinal properties. It has a longstanding history as an herbal remedy and a valuable addition to the diet. Previous research indicates that nettle possesses antioxidant, anti-inflammatory, anti-carcinogenic, and anti-aging properties [6,7]. Numerous studies have associated *Urtica dioica* extract with maintaining and enhancing cognitive performance [8,9]. Additionally, specific natural components within the extract, such as carvacrol, have been shown to regulate dopamine and serotonin levels in the hippocampus and prefrontal cortex, offering neuronal protection against damage from focal cerebral ischemia/reperfusion [10,11,12].

Although rodents have been the models of choice and have significantly contributed to our understanding of developmental neurotoxicity, experiments using large numbers of rodents are time-consuming and expensive and raise ethical concerns [13]. Using alternative non-mammalian animal models may relieve some of these pressures by granting large numbers of subjects for testing while reducing expenses and minimizing the use of mammals.

During the past decade, teleost fish have been introduced as successful vertebrate models in scientific research [14,15,16,17], emphasizing zebrafish species (*Danio rerio*) [18,19]. Zebrafish offer multifaceted advantages in developmental neurotoxicity testing, as seen in the concordance between zebrafish and human neurodevelopmental pathways reported in many studies [5,20,21]. Beyond this concordance, the conservativeness in protein and disease processes between humans and zebrafish further enhances their utility. Consequently, drugs exhibiting efficacy in humans exert similar effects on zebrafish, targeting the same biological pathways [22]. Moreover, the adaptability of zebrafish extends to their capability to absorb a diverse range of compounds from their surrounding media. This unique feature makes them particularly well suited for comprehensive drug screens [22]. Thus, the advantages of zebrafish underscore their significance in advancing research, particularly regarding developmental neurotoxicity and drug screening.

Considering the aforementioned data, we aimed to explore, for the first time, the impact of an ethanolic extract from *Urtica dioica* on mitigating the toxic repercussions induced by chlorpyrifos—an organophosphate insecticide with broad-spectrum effects—on both the developmental progress and neurobehavioral performance of zebrafish larvae.

## 2. Results

### 2.1. DI-HRMS Analysis

Based on the obtained mass spectra with DI-HRMS, which were collected through positive electrospray ionization (ESI+) and negative electrospray ionization (ESI−), as well as the molecular formula and unsaturation degree (RDB—Ring and Double Bond) values, various chemicals were identified in the ethanolic extract of *Urtica dioica* (UDE) and compared to data from the literature. This approach allowed for the identification of eight compounds. Concerning the ESI+ mode, five molecules were identified (isoledene, hexadecanethiol, heptadecenoic acid, ethylene glycole, and bornyl acetate). On the other hand, in the ESI− mode, three molecules were identified (oxo-octadecadienoic acid, esculin, and p-Coumaric acid) (Table 1).

### 2.2. Hatching and Survival Rates

**Figure 1:**
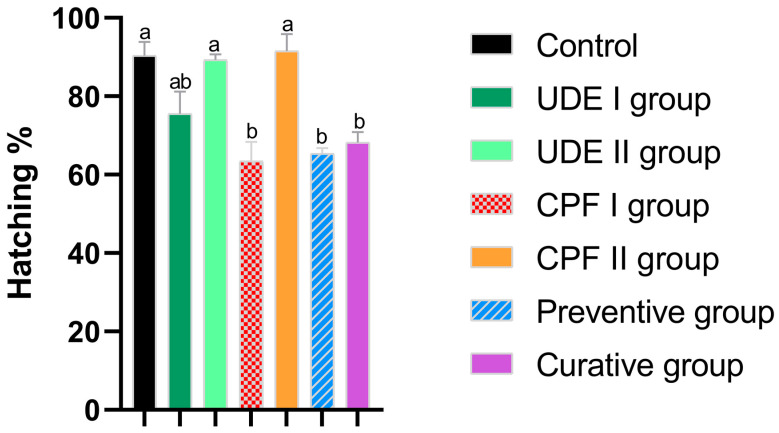
The hatching success rate at 78 hpf of the larvae from the different experimental groups. The data are expressed as the mean ± SD. The different lowercase letters indicate a significant difference between the experimental and control groups (Bonferroni, *p* < 0.05).

The hatching rate was monitored in the different groups until 72 h post-fertilization (hpf), which is the normal hatching period, and the total hatching rate was calculated. In the control condition, the hatching rate was around 90.3%. After a one-way ANOVA followed by Bonferroni correction, we recorded a 15% reduction in the hatching rate in UDE I compared to the control group; however, this difference was insignificant. In the UDE II I group, however, the observed difference in the hatching rates compared to the control groups was almost negligible and did not reach statistical significance. In the CPF I group, the amount of hatched larvae was almost 27% less than that in the control group, and the delta hatching rate was statistically significant. Furthermore, in the two experimental groups receiving both UDE and CPF, the hatching rate was considerably diminished compared to the baseline group but slightly higher than that reported in the CPF I group (Figure 1).

**Figure 2:**
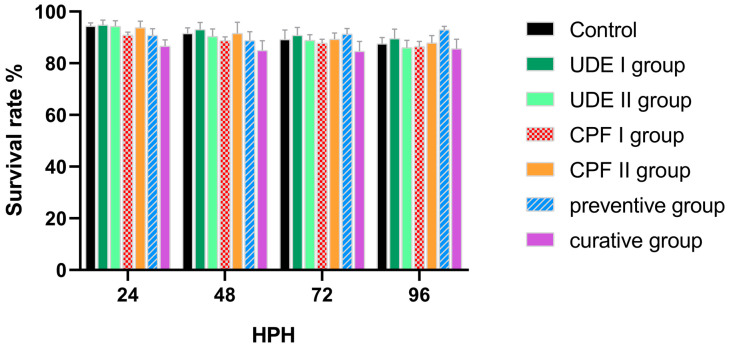
The survival rate at 24, 48, 72, and 96 hpf of larvae from the different experimental groups. The data are expressed as the mean ± SD. No significant difference between the experimental and control groups was reported (one-way ANOVA, Bonferroni, *p* < 0.05).

The survival rate, however, was monitored daily, and the UDE slightly increased the survival rate compared to the control group. This effect was noticeable in both the early treatment (UDE I) and the late treatment (UDE II) groups. Additionally, the toxic effect of the CPF detected in the hatching scoring was less evident, and the CPF I group had a lower survival rate compared to the CPF-free groups (control, UDE I, and UDE II). Still, no significant difference between the different groups was reported for the survival rate (Figure 2).

### 2.3. Teratology Screening

At five dpf, the larvae were assessed for morphological deformities. The morphological scoring consisted of evaluating the morphology of the different anatomical structures. 

**Figure 3:**
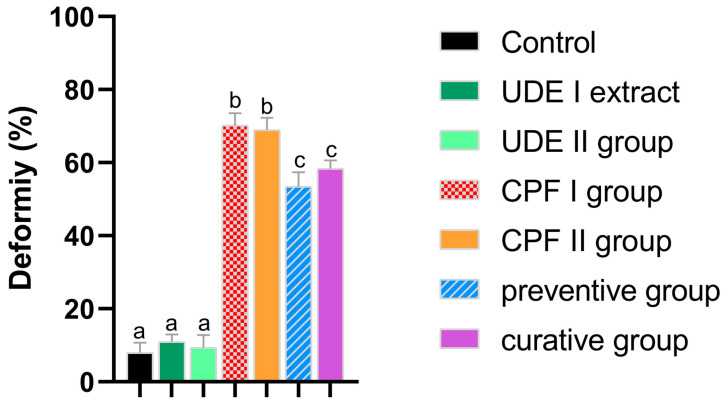
The deformity rate between 24 and 96 hpf of larvae from the different experimental groups. The data are expressed as the mean ± SEM. The different lowercase letters indicate a significant difference between the experimental and control groups (one-way ANOVA, Bonferroni, *p* < 0.05).

The UDE exposed the groups; thus, UDE I and UDE II had deformity rates that were comparable to that of the control group. The larvae exposed to CPF in both CPF I and CPF II, however, showed a significant increase in the percentage of individuals with morphological deformations. In both the preventive and curative groups, the deformation incidence was significantly lower than that of the CPF groups (Figure 3).

**Figure 4:**
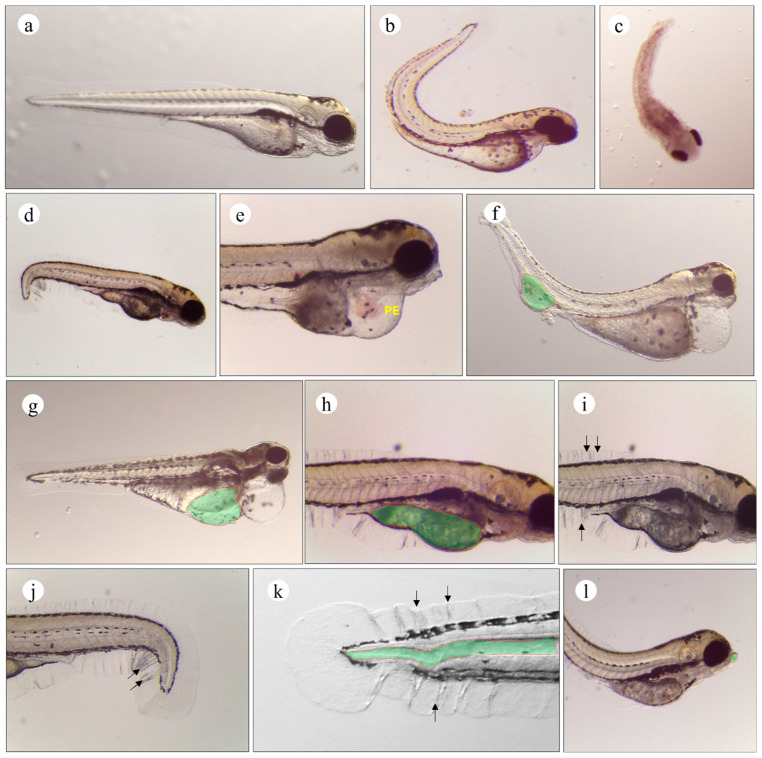
Morphological malformations observed in larval zebrafish exposed to CPF and UDE. (**a**) Morphology of normal zebrafish larva. (**b**) Lordosis. (**c**) Kyphosis. (**d**) Scoliosis. (**e**) Pericardial edemata. (**f**) Blood pooling at tail artery and pericardial edemata; (**g**) yolk edema and pericardial edemata. (**h**) Reduced yolk resorption. (**i**,**j**) Fin erosion. (**k**) Notochord deformation at caudal region. (**l**) Structural deformation of jaw. Black arrows indicate eroded fins.

Among the deformities registered during the experiment, there were deformities affecting the spinal cord, and three types of spinal curvatures could be differentiated, namely (1) the inward curving of the spine (or lordosis) (Figure 4b), (2) the outward curving of the spine (or kyphosis) (Figure 4c), and (3) the sideways curving of the spine (or scoliosis) (Figure 4d). Other than the spinal cord deformities, CPF induced pericardial edemata, which resembles an enlarged pericardium or distended thin-walled cavities surrounding the heart (Figure 4e). In other cases, pericardial edema was often associated with the pooling of blood in the caudal ventral region of the trunk (Figure 4f).

Morphologically discernible yolk alterations were identified starting from 48 hpf. Those deformities included yolk edemata (Figure 4g), which may develop into cavities within the yolk itself or concentrate in the periphery underneath the yolk sac. When healthy embryos began to deplete their nutrient resources, some larvae showed reduced yolk resorption (Figure 4h). Moreover, less frequent morphological alterations, including fin erosion (Figure 4i,j), deformation of the notochord (Figure 4k) with variable degrees of severity, and craniofacial malformations, including the lengthening of the lower jaw (Figure 4l), were encountered in a few larvae.

### 2.4. The Effects of UDE on the Brain-Derived Neurotrophic Factor Expression in ‘Larvae’ Telencephalon

**Figure 5:**
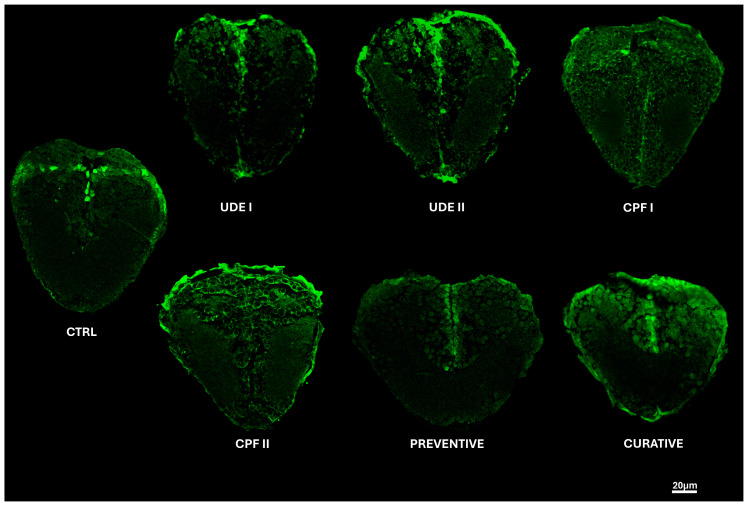
Bdnf immunolabeling in the telencephalon of the control, UDE I, UDE II, CPF I, CPF II, curative, and preventive groups. Scale bar: 20 µm.

In the control and UDE groups, the immunofluorescence positivity of the brain-derived neurotrophic factor (bdnf) was detected in the cytoplasm and nerve cell nuclei (Figure 5). In the CPF, preventive, and curative groups, the immunofluorescence positivity of bdnf was also reported in intercellular areas of neurons (Figure 5).

**Figure 6:**
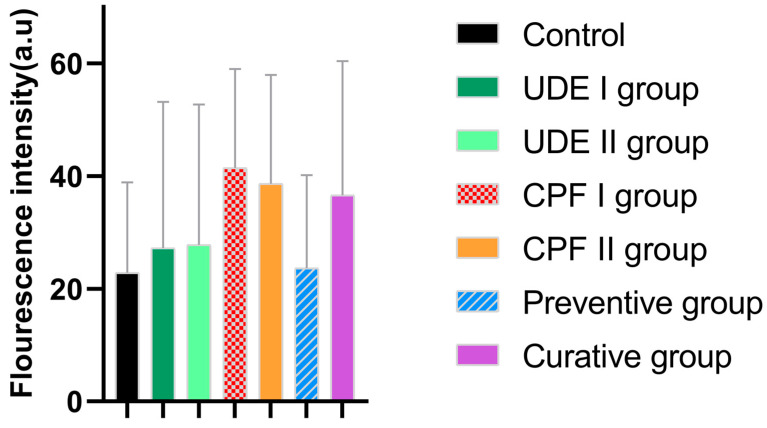
The quantification of the Bdnf immunolabeling mean intensity in the ‘larvae’ telencephalon. The data are expressed as the mean ± SEM. No significant differences between the experimental groups were reported (Kruskal–Wallis H, *p* < 0.05).

Bdnf immunolabeling in the in UDE I and UDE II groups was slightly higher than that of the control group, but the differences were still statistically insignificant (*p* ˃ 0.05). Moreover, both CPF groups, CPF I and CPF II, and the curative group exhibited stronger and more diffuse signals of bdnf antigens than the Ctrl and UDE groups, and the difference was not statistically significant either (*p* ˂ 0.05). Additionally, the preventive group showed a bdnf expression level comparable to those of the control and UDE groups (Figure 6).

### 2.5. Locomotor Activity

**Figure 7:**
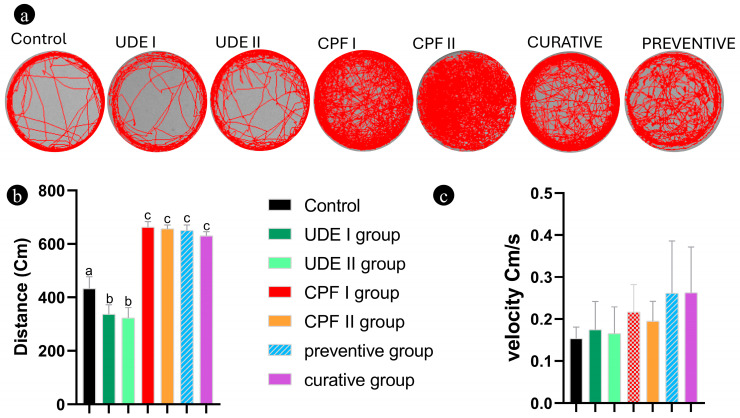
The locomotor behaviors of 96 hpf zebrafish larvae after exposure to CPF and UDE for two hours. (**a**) The records of larval motion trail. (**b**) The free swimming distance during a 120 min period (Bonferroni, *p* < 0.01). (**c**) The free swimming speed during a 120 min period with visible light (Bonferroni, *p* < 0.01). The data are expressed as the mean ± SEM of three replicates (10 larvae per replicate). The different lowercase letters indicate a statistically significant difference between the experimental groups at *p* < 0.01.

The records of the larval motion trail indicated that CPF exposure resulted in notable behavioral changes in the emotional trail and swimming distance. Indeed, these neurotoxic effects included irregular swimming (Figure 7a) and increased locomotor activity in the four groups exposed to CPF (CPF I, CPF II, preventive, and curative groups) compared to the control and UDE groups (Figure 7b,c). Moreover, the UDEI and UDE II larvae showed decreased motility compared to the Ctrl group in terms of swimming distance but not speed. Larvae exposed to UDE preceding or following CPF exposure and, therefore, those of the preventive and curative groups, have slightly reduced motility compared to the larvae in the CPF ‘group’. Still, this difference was not significant (Figure 7c). In both UDE I and II, and in CPF I and CPF II, there was no statistically significant difference between the groups that were treated early (4/6-48 or 4/6-72 hpf) and late (3-5 or 2-5 DPF) (Figure 7b,c).

## 3. Discussion

Chlorpyrifos is a broad-spectrum organophosphorus insecticide that exhibits high toxicity to non-target organisms. Human exposure has raised concerns regarding its toxicity to the developing nervous system. Indeed, since it is from the same family of chemicals as the sarin nerve gas agent, CPF functions by attacking the central and peripheral nervous system, inhibiting the enzyme activity of acetylcholinesterase and butyrylcholinesterase, the neurotransmitter choline ester catalyzers, leading to the accumulation of acetylcholine and cholinergic hyperstimulation [3,4,23].

Studies on experimental animal models confirmed that exposure to CPF during critical stages of brain development can cause persistent neurobehavioral deficits and altered locomotor activity even at doses that do not elicit acute cholinergic toxicity or a significant downregulation of cholinergic receptors [24,25,26]. Moreover, they suggested that developmental neurotoxicity could be induced by altering neuronal connectivity in the developing brain [27,28].

During the current study, the chlorpyrifos-treated groups received a final concentration of 1 mg/L. This concentration was chosen to be high but under the LC50 concentration of 1.520 mg/L, with a 95% confidence limit of 1.26–1.82 mg/L, which was already defined by a previous study [29]. The chosen concentration of 65.78% LC50 ensured a chlorpyrifos-induced toxicity phenotype.

In their study, Jiang et al. exposed zebrafish to 0.5% LC50 of chlorpyrifos (7.6 µg/L) for 35 days [29]. Although the aforementioned dose was smaller than that used in the current study, Jiang’s team demonstrated a significant bioaccumulation of chlorpyrifos in the intestinal tract and brain, with a smaller accumulation observed in the gills and muscles. CPF bioaccumulation in different tissues has been recorded in other fish species. Indeed, a study on largemouth bass (*Micropterus salmoides*) juveniles exposed to 4 µg/L of CPF (1/5 of 96 h LC_50_) was conducted, and the CPF bioaccumulation in muscle tissues was recorded. The differential accumulation patterns of chlorpyrifos in zebrafish tissues could be explained by the induced oxidative stress and the inhibition of some detoxifying enzymes, namely carboxylesterase and cytochrome P450s, with their activity exacerbating the pesticide metabolism process [30]. Moreover, zebrafish larvae are likely to have a higher bioaccumulation of pesticides compared to adults primarily due to their less developed detoxification systems and higher surface area-to-volume ratio. Due to the evidence of the high bioavailability of chlorpyrifos, even at low exposure concentrations, the authors decided not to assess its bioaccumulation in zebrafish larvae in the current study, avoiding the need to sacrifice a significant number of larvae in each group for this assessment.

*Urtica dioica* has a longstanding history as an herbal remedy and a valuable addition to the diet. Indeed, previous research indicates that nettle possesses antioxidant and anti-inflammatory properties and has great potential for maintaining and enhancing cognitive performance and neuronal protection against damage from focal cerebral ischemia/reperfusion [7,8,9,10,12,13].

Those potential effects are conferred to *Urtica dioica* by its content on natural compounds of great interest for neuronal development and protection, including those revealed by the current study. Other than *Urtica dioica*, isoledene was encountered in the oleo-gum resin of Ceylon ironwood (*Mesua ferrea*) [31] and in the essential oils of anise (*Pimpinella anisum* L.), fennel (*Foeniculum vulgaris* M.) [32], and false daisy (*Eclipta prostrata* L.) [33]. As reported in another study, isoledene can elevate the levels of caspases-3/7, −8, and −9 and ROS [31]. Bornyl acetate, however, was previously identified in Pinus family essential oils [34], rosemary essential oil [35], and *Ferula ovina* (Boiss.) F. aerial ‘parts’ essential oil [36]. It could alleviate demyelinating diseases via its anti-inflammatory effect, inhibiting mitogen-activated protein kinases and nuclear factor-kappa B pathways and reducing the mobilization of CD4+ T, Th1, and Th17 cells [37,38]. Heptadecanoic acid has the same effect, as it is known for its potential to inhibit inflammation by inhibiting the NF-κB pathway [39].

Oxo-octadecadienoic acid, a natural agonist with antioxidant and anti-inflammatory effects [40,41], can activate PPARα in the brain, and it is a promising target in treating neurodegenerative disorders and neuroprotection [42,43].

Esculin is another natural compound identified in UDE in the current study. It is a well-studied coumarin component known for its various pharmacological properties, including the induction of immunomodulatory, antioxidant, and anti-inflammatory effects [44], via the MAPK pathway [45]. Additionally, esculin is known for its capacity to ameliorate behavior and recognition memory in experimental diabetic nephropathy [45]. Furthermore, in an in vitro study using the human neuroblastoma SH-SY5Y cell line, Zhao et al. found that esculin inhibited the release of cytochrome c and apoptosis-inducing factor, suggesting its use in the therapeutic strategy for the treatment of progressive neurodegenerative diseases [46].

P-Coumaric acid is another interesting natural compound that has been identified in UDE. Its antioxidant and anti-inflammatory properties have already been recorded in many studies. Its protective effect on neuroinflammation, cognitive impairment, and neuronal apoptosis was attributed to its anti-apoptotic, antioxidant, AChE inhibitory, and anti-inflammatory activities and increased CREB phosphorylation [47,48].

The current investigation assessed the potential beneficial effect of *Urtica dioica* ethanolic extract against chlorpyrifos-induced toxicity by evaluating the hatching and survival rates, teratogenic effects, and Bdnf expression in ‘larvae’ telencephalon as well as the behavior of zebrafish larvae.

Hatching was recorded daily in the different groups from 48 to 72 hpf, which is the physiological hatching period [49]. In the control condition, the hatching rate was around 90.3%, which is comparable to that reported in previous zebrafish studies [50,51]. The UDE did not exhibit a significant effect on hatching, whereas exposure to CPF resulted in altered hatching. This observation is in contradiction with the findings of Yu et al., who demonstrated that CPF altered embryonic incubation and that the 1 mg/L CPF group showed a hatching rate of 80% at 48 hpf compared to <15% in the control group [52]. Still, the current study’s findings are in accordance with the findings of Jin et al., who found that CPF reduced hatchability in a dose-dependent manner [53]. Moreover, even when combined with the UDE, CPF significantly delayed hatching.

The delayed hatching that was observed may stem from the embryos’ inability to break out the chorion in some eggs. This hypothesis finds support in the detrimental impact of CPF on locomotor activity, which was previously reported in zebrafish and mammals [54,55]. Another hypothesis could be the capacity of CPF to trigger the inhibition of the enzyme responsible for breaking down the chorion: Hatching Enzyme 1. Thus, the intact chorion resists the larvae’s first spontaneous movements and keeps them restricted.

The toxic effect of CPF detected in the hatching scoring was less evident in the survival rate. The CPF effect reported in the survival rate was compared to that recorded in a previous study with a 1 mg/L exposition concentration [52].

The teratogenic scoring of zebrafish larvae consists of assessing the morphology of the different anatomical structures of larvae. The CPF group exhibited a notable rise in the recurrence of morphological deformity compared to the control group, thus affirming the well-established teratogenic effects of CPF [30,53]. In the preventive group, the incidence of deformation was lower than that of the CPF group, reflecting a valid preventive effect of *Urtica doica* extract. Although the incidence of those deformities in the curative group was lower than that observed in the CPF group, the difference was not statistically significant, thereby abolishing the curative potential of the UDE. This observation may be attributed to the potential of the UDE to target the mechanisms associated with teratogenicity development compared to its lesser or negligent capacity to reverse established conditions.

Among the deformities registered during the experiment, spinal cord deformities, pericardial edema, and discernible yolk alterations were identified starting from 48 hpf. Those deformities were consistent with those typically associated with CPF exposure, which were documented in previous studies at even lower exposition concentrations (400, 600, and 800 μg L^−1^) compared to that used in the current study [56], as well as in the case of combined pesticide exposure experiments [57]. Late and early CPF exposure did not affect the incidence of deformity. Furthermore, no group-specific deformities were reported. This observation could be explained by the capacity of CPF, which was administered starting from 72 hpf, to induce broad metabolic disruptions which, in turn, interrupted the physiological increase in metabolite concentrations reported in zebrafish larvae between 48 and 72 hpf [58,59].

The edema formation in embryos reported in the larvae exposed to CPF could be explained by osmoregulation failure associated with gill damage. Indeed, many studies have substantiated the highly toxic effect of CPF on gill cells across various species [60,61,62].

During the first 96 h of development, the yolk sac plays an important role as the main source of nutrients. In normal conditions, yolk sac resorption is evident at approximately 120 hpf [63]. In the current study, some cases of reduced yolk resorption were recorded while healthy embryos began to deplete their nutrient resources, which was interpreted as a symptom of embryonic malabsorption syndrome. To the authors’ knowledge, this observation is unique since it was not reported before in studies using CPF on zebrafish or another organism model.

Yolk sac and pericardial edema, defined as ‘blue sac syndrome’, was among the recurrent developmental toxicity pathology induced by CPF exposition. This pathology has been demonstrated to be caused by many organophosphates [64,65,66] and other toxicants [67] in zebrafish embryos. The wide range of chemicals that induce yolk sac and pericardial edema highlight that both deformities are sensitive toxicological outcomes for embryonic evaluation.

Moreover, spinal cord deformities; craniofacial malformations, including the lengthening of the lower jaw; as well as fin erosion observed in the CPF-exposed groups may be attributed to CPF interference with the ossification process [68] or the downregulations of the pkt7 gene [69].

The evaluation of the expression pattern of BDNF conducted on zebrafish larvae’s telencephalon highlighted the stronger and more diffuse signals for bdnf antigens in the CPF-exposed and curative groups compared to the Ctrl and UDE groups, although these differences were not statistically significant. These findings are in accordance with the results of Özdemir et al., who proved that in adult zebrafish exposed to chlorpyrifos or other common pesticides, namely cypermethrin, deltamethrin, and imidacloprid, an intensive upregulation of bdnf was induced in the tissues exposed to pesticides compared to the control group [70]. The higher bdnf expression in the CPF-exposed groups reflects its effect on scavenging the toxic effects of CPF. Indeed, bdnf is known as a promoter of dendritic genesis, ensuring nerve cell survival, it plays key roles in growth, differentiation, and synaptic plasticity [71,72], and it reduces neuronal apoptosis [73]. Bdnf immunolabeling in curative and preventive groups was slightly lower than that of the CPF group (*p* ˂ 0.05). A CPF-UDE physiological antagonism could explain these findings. Indeed, unlike CPF, UDE is known for its potential to increase BDNF, among the other neurotrophin levels in rats [74] and mice [75]. Moreover, the bdnf fluorescence intensity was comparable between the control and the preventive groups, suggesting that UDE has a protective effect against CPF neurotoxicity. Indeed, Urtica dioica extract’s strong antioxidant capacity and potential to reduce myelin degradation and improve brain histopathology are well documented [76,77].

Many studies defining CPF as an acetylcholine esterase inhibitor that interferes with organism neurobehavioral development have been published during the past decade [4,5,78,79]. CPF exposure was reported to affect behavior in rodents [80,81] and different fish species, namely zebrafish [82], mosquito fish [83], common carp [84], and spotted snakehead [85]. These results are stackable to the current study’s findings on zebrafish larvae. Indeed, the 96 hpf larval motion trail record indicated that CPF exposure resulted in notable behavioral changes in terms of the emotional trail and swimming distance. The decreased motility of UDE larvae compared to the Ctrl group was contradictory with the finding of DI Izunwanne et al., who reported in a recent study that a repeated administration of *Urtica dioica* enhanced locomotory behavior in mice [86].

## 4. Materials and Methods

### 4.1. Plant Collection and Extraction

*Urtica dioica* L. aerial parts specimens were collected from Bir Ali commune (Sfax Governorate) in eastern Tunisia in February 2022. The specimens were washed with distilled water, dried at room temperature for one week, and ground into powder. Twenty grams of the powdered specimens was poured with 70% ethanol and left at 37 °C for three days (maceration technique). The extract was filtered using Whatman filter paper, and then the filtrate was placed in a rotary evaporator (EYELA N1000, Tokyo, Japan) at 40 °C to eliminate ethanol.

### 4.2. Direct-Infusion High-Resolution Mass Spectrometry Analysis

The identification of UDE compounds was conducted using the direct-infusion high-resolution mass spectrometer (DI-HRMS) technique. The target analytes were determined in positive mode in the full mass scan function (*m*/*z* 100 to 900) using a Thermo Scientific™ system (Bremen, Germany) with a heated electrospray ionization source. The obtained results were processed using the Xcalibur 2.2 software. The samples were carried out by direct introduction at a flow rate of 10 μL·min^−1^. The exact theoretical mass (M) was determined from the formula in which one proton is added for positive mode [M-H+] and one proton is subtracted [M-H−] for negative mode. Moreover, the tentative assignment according to the literature, the elementary formula, the exact mass for two modes of ionization, and Ring and Double Bond (RDB) were determined for each compound.

### 4.3. Embryos Maintenance and Treatments

**Figure 8:**
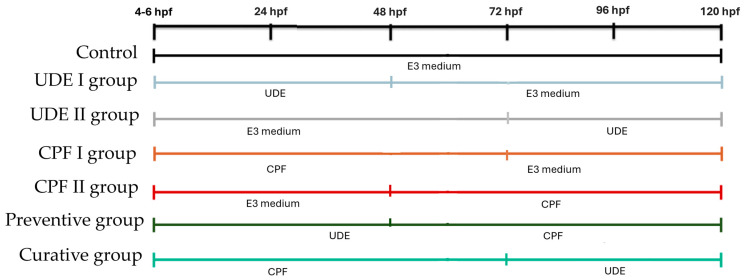
The schedule of the experimental conditions.

Wild-type 4–6 hpf embryos were examined under a stereo microscope, and unfertilized and dead embryos were removed. Embryos were distributed in 5 Petri dishes, with 20 embryos each, containing 15 mL of E3 medium or designated treatment solution. The embryos were maintained under standard laboratory conditions at a temperature of 27 ± 0.5 °C with a photoperiod of 14:10 (light/dark). The experiment was performed in triplicate, with each experimental group being assigned as follows (Figure 8):

(1) The control group with no treatment.
(2) The UDE I group, which was treated with UDE freshly prepared in E3 medium at a final concentration of 25 mg/L, starting at 4–6 hpf until 48 hpf was reached.
(3) The UDE II group, which was treated with UDE freshly prepared in E3 medium at a final concentration of 25 mg/L, starting at 72 hpf until the end of the experiment was reached.
(4) The CPF I group, which was treated with chlorpyrifos (campagnie, Sfax, Tunisia) from 4–6 hpf to 72 hpf, at a final concentration of 1 mg/L dissolved in E3 medium. This concentration was chosen based on LC_50_ = 1.520 mg/L, with a 95% confidence limit of 1.26–1.82 mg/L [29], which is moderately toxic to zebrafish larvae.
(5) The CPF II group, which was treated with chlorpyrifos (campagnie, Sfax, Tunisia) from 48 hpf to 120 hpf at a final concentration of 1 mg/L dissolved in E3 medium.
(6) The UDE+CPF preventive group in which UDE was maintained from 4–6 hpf to 48 hpf and CPF was administered from 48 hpf to 120 hpf.
(7) The CPF+UDE curative group in which CPF was maintained from 4–6 hpf to 72 hpf and UDE was administered at 72 hpf and maintained until 120 hpf.

The treatment solution and E3 medium were renewed daily to maintain the test concentrations of CPF and/or UDE. At 120 hpf, the embryos were euthanized using tricaine mesylate.

### 4.4. Teratology Assessment

Each day, viable embryos/larvae were counted in order to calculate the hatching and survival rates. Larvae were assessed for apparent morphological anomalies under a Leica M205C stereomicroscope (Leica, Milan, Italy). Zebrafish larvae were scored with respect to the recurrent teratogenic endpoints, namely the curvature of the body axis; structural malformation of the jaw, notochord, or fin; and edema in the heart, pericardial, and yolk sac regions. Stereomicrographs were taken using a Leica IC80 HD digital camera (Leica, Milan, Italy).

### 4.5. Tissue Processing and Immunofluorescence

After fixation on 4% PFA, larvae from the different groups were processed for paraffin wax embedding. They were then sectioned using Leica RM2135 microtome (Leica, Milan, Italy) at a thickness of 7 µm and thaw-mounted onto gelatin-coated microscope slides. Slides were dried for 24 h and then processed for immunofluorescence.

The immunofluorescence technique was used to evaluate the expression of BDNF in the larvae telencephalon sections as previously described [14]. In brief, sections were rinsed in Tris-HCl buffer (0.05 M, pH 7.5) containing bovine serum albumin and Triton-X 100. Nonspecific binding was blocked by covering slides with 25% fetal calf serum, after which sections were incubated overnight with the primary antibodies rabbit polyclonal anti-brain-derived neurotrophic factor (Cat. # AB1534SP, Merck Millipore, Burlington, MA, USA) (Table 2). The specificity of the anti-brain-derived neurotrophic factor (Cat. # AB1534SP, Merck Millipore, Burlington, MA, USA) was proven in previous studies [87,88]. After an appropriate rinse, incubation with the secondary antibodies for 90 min was performed (Table 2). Negative controls were carried out, barring the primary antibody, and immuno-labeling was wholly abolished. Finally, specimen immunolabeling was evaluated using a confocal laser scanning microscope (Zeiss LSM 700, Carl Zeiss Micro Imaging GmbH, Jena, Germany) with a META module.

### 4.6. BDNF Fluorescence Intensity at Telencephalon

To determine the expression of BDNF in the telencephalon, we calculated the corrected total fluorescence of the area of interest by employing the Freehand ROI tool of the Zen 2011 program (Zeiss blu edition, Carl Zeiss MicroImaging GmbH, Jena, Germany). The corrected total cell fluorescence was calculated by subtracting the background fluorescence from a minimum of 3 sections of 7 μm in thickness from at least three larvae per group.

### 4.7. Larval Behavior

By the end of the experiment, at 120 hpf, larvae were placed in individual wells of a 4-well transparent spot plate with 1 mL of E3 medium. After 10 min of acclimation in the dark, we recorded behavioral changes of zebrafish larvae using the DanioVisionTM observation system (Noldus, Wageningen, The Netherlands, Model: 17.0.1630) for 120 min. A behavioral assay was conducted in a temperature-controlled room at 26 ± 1 °C, and the light intensity was adjusted to 2412 lux.

Each zebrafish larva’s accumulated behavioral data activity was analyzed for three endpoints. The total distance traveled and the velocity were calculated using Ethovision^®^XT (Noldus, VA, USA).

### 4.8. Statistical Analysis

Statistical analyses were conducted and graphs were created using IBM SPSS Statistics for Windows version 22, (IBM Corp, Armonk, NY, USA) and GraphPad Prism version 8.0.1 for Windows (GraphPad Software, San Diego, CA USA). After confirming data normality using the Shapiro–Wilk test and homogeneity of variance by Levene’s tests, differences between the groups were analyzed using ANOVA followed by Bonferroni correction. When homogeneity of variance is violated, nonparametric Kruskal–Wallis test was used to compare treatment and control groups as an alternative to the parametric analysis of variance (ANOVA). Significance between groups was accepted when *p* ≤ 0.05.

## 5. Conclusions

Chlorpyrifos induced toxic effects on zebrafish larvae, including altered hatching, increased teratogenicity, and disrupted behavior. Moreover, the UDE exhibited promising protective properties against CPF-induced toxicity, as evidenced by its ability to counteract the teratogenic effects and preserve locomotor activity, which could be assigned to its natural antioxidant and anti-inflammatory compounds. Still, the UDE’s protective effect was modest for the hatching process. A potential antagonistic interaction between CPF and UDE for the Bdnf expression level was recorded. Overall, our findings underscore the potential of UDE as a protective agent against CPF-induced toxicity, even though further mechanistic studies are needed to elucidate its therapeutic mechanisms. This research contributes to the understanding of CPF-induced toxicity and offers insights into the potential of natural compounds as therapeutic agents to counteract the adverse effects of contaminants.

## Figures and Tables

**Table 1:** High-resolution accurate mass data, tentative assignment, formula, and Ring and Double Bond (RDB).

Tentative Assignment	Formula	[M-H] + Found	[M-H] − Found	RDB
Isoledene	C15H24	219.17525	-	4.5
Hexadecanethiol	C16H34S	256.13380	-	4
Heptadecenoic acid	C17H34O2	278.07987	-	8
Ethylene glycol	C18H38O4	318.30066	-	0
Bornyl acetate	C12H20O2	153.13930	-	2.5
Oxo-octadecadienoic acid	C18 H3003	-	293.17831	4.5
Esculin	C15 H1609	-	339.19858	7.5
p-Coumaric acid	C15 H1808	-	325.18317	7.5

**Table 2:** Antibodies used for immunohistochemical study.

	Antibody	Dilution
Primary antibodies	Anti-brain-derived neurotrophic factor (Cat. # AB1534SP, Merck Millipore, Burlington, MA, USA)	[1:100]
Secondary antibodies	Goat anti-rabbit IgG (H + L) cross-adsorbed secondary antibody, Alexa Fluor™ 594 (Thermo Fisher Scientific, Chino, CA, USA, Cat. # A-11012)	[1:100]

## Data Availability

Data is contained within the article.
